# Influence of adding fibrolytic enzymes on the ruminal fermentation of date palm by-products

**DOI:** 10.5194/aab-62-1-2019

**Published:** 2019-01-21

**Authors:** Khalil Abid, Jihene Jabri, Yves Beckers, Hela Yaich, Atef Malek, Jamel Rekhis, Mohamed Kamoun

**Affiliations:** 1 Food and Animal Nutrition Service, National School of Veterinary Medicine Sidi Thabet, University of Manouba, Manouba, Tunisia; 2 Institut Supérieur Agronomique de Chott-Mariem, University of Sousse, Sousse, Tunisia; 3 Laboratories Precision Livestock and Nutrition, Gembloux Agro-Bio Tech, University of Liège, Liège, Belgium

## Abstract

This study was conducted in order to assess the influence of four doses (0, 0.5, 1, and 2 mg (g dry matter)${}^{-1}$ of commercial fibrolytic enzymes (MAXFIBER-I${}^{®}$, SHAUMANN GmbH, Wahlstedt, Germany) on in vitro fermentation of date palm (*Phoenix dactylifera*) by-products: date kernels, wasted dates, floral stems, and palm fronds. Rumen contents were obtained from two non-lactating Holstein cows. Enzyme supplementation to by-products was carried out 12 h prior to incubation.

Compared to the control, the enzymatic supplementation quadratically increased the extent but not the gas production rate of date kernel fermentation. Indeed, the potential gas production increased notably by 14.8 % with the lowest enzymes dose following recorded gas production after 48, 72, and 96 h of incubation. The estimated organic-matter digestibility, metabolisable energy, and total volatile fatty acids in the incubation fluid tended to be increased with the lowest dose by 7.8 %, 8.4 %, and 13.9 % respectively. For the wasted dates, this feed additive tended to linearly increase the gas production rate of fermentation with the highest dose. On the other hand, this supplementation had no effect on the ruminal fermentation of the floral stems and palm fronds. The exogenous fibrolytic enzymes were more effective on fibrous but not on lignified date palm by-products.

## 1 Introduction

The date palm tree (*Phoenix dactylifera*) is the principal source of food for the human population of oases. By-products of this tree are used in ruminants' nutrition by local breeders (Genin et al., 2004). In recent years, many studies evaluated the chemical composition and nutritive value of these by-products. Indeed, date kernels (DKs), floral stems (FSs), and palm fronds (PFs) are characterised by high levels of lignocellulose content, low protein content, low digestibility, and low palatability. Wasted dates (WDs) are characterised by low protein and lignocellulose content (Genin et al., 2004; Kholif et al., 2015; Boufennara et al., 2016; Azzaz et al., 2017). Many technologies have been employed to increase the nutritive values of poor-quality roughage. Recently, many studies evaluated the effect of the addition of fibrolytic enzymes to ruminants' nutrition (Beauchemin and Holtshausen, 2010). This treatment increased fibre digestibility (Elghandour et al., 2013; Gandra et al., 2017). The mechanism of this increase is due to the partial hydrolysis of neutral detergent fibre and acid detergent fibre (Nsereko et al., 2000; Wang et al., 2001; Giraldo et al., 2008; Krueger et al., 2008a). This hydrolysis provides monosaccharides (Nsereko et al., 2000), which promote rumen microorganism proliferation and colonisation on the substrate (Nsereko et al., 2000, 2002; Wang et al., 2001). Nevertheless, other studies reported that exogenous fibrolytic enzymes had negligible effects (Dean et al., 2013; Peters et al., 2015). The beneficial impact of the exogenous fibrolytic enzymes based on xylanase and cellulase depends on several factors, such as the types of substrate (Elghandour et al., 2013; Diaz et al., 2013), doses of the enzymatic preparation (Diaz et al., 2013; Elghandour et al., 2013, 2016), and methods of application of this feed additive (Krueger et al., 2008b; Elghandour et al., 2016). Some researchers have evaluated the effect of adding the exogenous fibrolytic enzymes to date palm by-products. Kholif et al. (2015) and Azzaz et al. (2017) studied the effect of exogenous fibrolytic enzyme supplementation on date kernels. These two studies showed that the addition of exogenous fibrolytic enzymes improved nutrient digestibility of date kernels for sheep and goats and increased the milk production of goats. Therefore, the aim of this study was to determine the influences of commercial fibrolytic enzymes (MAXFIBER${}^{-}$I^®^) on in vitro fermentation of date palm by-products.

## 2 Material and methods

### 2.1 Sampling and chemical analysis

Date palm (*Phoenix dactylifera* L.) by-products from a local variety (*Chedakh*), including DK, WD, FS, and PF were collected from El Hamma Oasis (Gabès region, southeast Tunisia). These by-products were pre-dried at 55 ${}^{\circ }$C for 48 and then ground to 1 mm using a Cyclotec 1093 Sample Mill (Tecator, Höganäs, Sweden). DK, WD, FD, and PF were analysed for their dry matter (DM) (Method 967.03), crude protein (CP) (Method 981.10), crude fibre (CF) (Method 935.53), ether extracts (EEs) (Method 920.29), ash (Method 942.05) calcium (Method 935.13), and phosphorus (Method 972.22). These by-products were analysed according to AOAC (1990). Neutral detergent fibre (NDF), acid detergent fibre (ADF), and acid detergent lignin (ADL) were analysed by the method of Van Soest et al. (1991) using a fibre analyser (type ANKOM${}^{220}$, ANKOM Technology, Macedon, NY, USA). The condensed tannin was analysed by weighing 200 mg of by-products into a 50 mL flask. The feed sample was extracted with 10 mL aqueous acetone 70 % in an ultrasonic bath for 2h, and the contents were centrifuged for 20 min at 3861 rpm and 4 ${}^{\circ }$C. The supernatant was collected. In total, 0.5 mL of this tannin extract was treated with 3 mL of butanol HCl in the presence of 0.1 mL of the ferric ammonium sulfate. The absorbance was 550 nm (Makkar, 2000). Total sugars in wasted dates were measured following the method of Dubois et al. (1956). Each analysis was carried out in three replications.

The nitrogen-free extract (NFE), organic matter (OM), hemicellulose, and cellulose were calculated, with this equation:

1NFE=100-(NDF+CP+EE+ash)(NRC,2001).2OM=100-ash(AOAC,1990)3Hemicellulose=NDF-ADF(VanSoestetal.,1991)4Cellulose=ADF-ADL(VanSoestetal.,1991)

NFE, NDF, CF, EE, OM, ash, hemicelluloses, ADF, cellulose, and ADL are expressed as % DM.

### 2.2 Enzymatic activity

The commercial exogenous fibrolytic enzymes (EFEs) used, produced from strains of *Aspergillus niger*, *Aspergillus tubingensis*, *Aspergillus orzyae*, *Aspergillus sojae*, and *Neurospora intermedia* (MAXFIBER${}^{-}$I^®^, SHAUMANN GmbH, Wahlstedt, Germany) were analysed for their xylanase, endoglucanase, and exoglucanase activities in three repetitions, according to the procedure of Bailey et al. (1992) and Wood and Bhat (1988). These activities were determined at a pH of 6.6 and a temperature of 39 ${}^{\circ }$C. These reflect the normal rumen conditions of the dairy cow. Xylanase activity was tested using (1 %, w/v) of oat spelt xylan (catalogue no. X-0627, Sigma Chemical Co., St Louis, MO, USA) as a substrate. Endoglucanase activity was tested using 1 % (v/v) medium viscosity carboxymethylcellulose sodium salt (catalogue no. C-4888, Sigma Chemical Co., St Louis, MO, USA) as a substrate. Exoglucanase activity was examined using cellulose (catalogue no. S-3504, Sigma Chemical Co., St Louis, MO, USA) as a substrate. According to the manufactures, MAXFIBER${}^{-}$I${}^{®}$ is a fibrolytic enzyme powder containing xylanase, endoglucanase, and exoglucanase activities.

### 2.3 Enzyme treatment

The four by-products were sprayed with an EFE at 0 (control), 0.5 (low), 1 (medium), and 2 mg (g DM)${}^{-1}$ (high), 12 h prior to starting the incubation. This low dose was recommended by the manufacturing company.

### 2.4 Experiment 1: pre-incubation effects

The EFE was used to solubilise the OM during the pre-interaction period with palm by-products, prior to incubation with rumen fluid, following the procedure of Colombatto et al. (2003) and Elwakeel et al. (2007). Samples of 1 g DM of each substrate supplemented with the EFE with an adequate dose were weighed into glass flasks; then 100 mL of distilled water was added. This mixture was stored for 12 h at the ambient temperature. After incubation, residues were recovered by filtration with Whatman 541 filter paper (Maidstone, Kent, England). The organic-matter losses were determined according to the methods described by AOAC (1990).

### 2.5 Experiment 2: in vitro incubation

Samples of 0.2 g DM of each substrate mixed with an adequate dose of EFE were pre-weighed into 120 mL serum bottles. Each sample was incubated in triplicate in two runs for each treatment. Rumen contents were collected from two non-lactating fistulated Holstein cows, before morning feeding. These cows were fed twice daily with 2 kg of commercial concentrate and pre-grown grass ad libitum. Rumen contents were filtered with two layers of cheesecloth under a continuous flow of ${\mathrm{CO}}_{2}$ in the water bath at 39 ${}^{\circ }$C. The rumen inoculum was diluted (1:2, v/v) with the buffer solution (Menke and Steingass, 1988). Thirty millilitres of the incubation inoculum were added to each serum bottle, which was closed with a rubber stopper. The bottles incubated in a shaking water bath for 96 h at 39 ${}^{\circ }$C. Three blank samples, without substrate, were also used to correct the gas production. Gas pressure was tested at 2, 4, 6, 8, 12, 24, 48, 72, and 96 h by a pressure transducer (model PX4200-0100GI; Omega Engineering, Inc. Laval, QC, Canada), attached to a visual display transducer (Data Tracker 200, Data Track Process Instruments Ltd, Christchurch). After each measurement, the gas produced was released. Net gas pressure, for each treatment, was calculated by subtracting the gas production from blank bottles. The gas pressure was converted into the volume as follows:

5 $$G_{v}\left(t\right)=\frac{G_{P}\left(t\right)\times (V_{f}-V_{i})}{P_{\mathrm{atm}}},$$

where $G_{v}$ is the volume of gas produced at an incubation time t (mL), $G_{P}$ is the gas pressure recorded at incubation time t (bar), $V_{f}$ is the volume of the bottle (mL), $V_{i}$ is the volume of inoculum added at the start of incubation (mL), and $P_{\mathrm{atm}}$ is the atmospheric pressure (bar).

The kinetics parameters of gas production were determined with the non-linear model (Proc NLN) option of SAS (2001) following Ørskov and McDonald (1979):

6 $$Y_{\left(t\right)}=B(1-e^{-Ct}),$$

where Y is the cumulative volume of gas produced (mL (g DM)${}^{-1}$), B is the potential gas production (mL (g DM)${}^{-1}$), C is the gas production rate (mL (h)${}^{-1})$, and t is the incubation time (h).

The time $T_{1/2}$, at which the gas production is half the potential gas production, was calculated from kinetic results as

7 $$T_{1/2}=\frac{\mathrm{ln}\;2}{C}.$$

The metabolisable energy (ME) values, organic-matter degradability (OMD) contents, and volatile fatty acids (VFAs) contents in the incubation fluid were estimated:

ME=2.2+0.136×GP+0.0057×CP8(MenkeandSteingass,1988)OMD=14.88+0.889×GP+0.45×CP+0.0651×Ash9(MenkeandSteingass,1988)10VFA=0.0222×GP-0.00425(Getachewetal.,2002),

where VFA is given in mmol (200 mg DM)${}^{-1}$, ME in MJ (kg DM)${}^{-1}$, OMD in %, CP in % DM, EE in % DM, and ash in % DM and GP is the net gas production (mL) from 200 mg after 24 h of incubation.

### 2.6 Statistical analysis

The different data of experiments 1 and 2 were analysed using a statistical factorial model (4×4) to assess the effect of four doses of exogenous fibrolytic enzymes (0, 0.5, 1, and 2 mg (g DM)${}^{-1})$ and four date palm by-products (DK, WD, FS, and PF), with three replicates per treatment. Then, this experiment was repeated twice. Mean values of each individual sample from each run within each treatment were used as the experimental unit. Data from the experiment were performed by using Proc GLM of l SAS (2001), with the following model:

11 $$Y_{ijk}=\mu +D_{i}+S_{i}+(S\times D{)}_{ij}+e_{ijk},$$

where $Y_{ijk}$ is the observation of ith doses and jth by-product, μ is the mean, $D_{i}$ is the enzyme dose (i=0, 0.5, 1, and 2 mg (g DM)${}^{-1})$, $S_{j}$ is the substrate (j= DK, WD, FS, and PF), S×D is the interaction between the enzyme doses and substrates, and $e_{ijk}$ is the experimental error. The orthogonal contrast was used to test linear and quadratic effects of dose for each date palm by-product. The orthogonal coefficients for unequally spaced treatment doses of the enzyme were determined by using PROC IML (SAS, 2001). The Duncan test was used to test the significant difference between the mean of data (Duncan, 1955). Significance was declared at P<0.05. Differences with 0.05<P<0.1 were accepted as representing tendencies to differ.

## 3 Results

### 3.1 Chemical composition

FS and PF are characterised by high levels of lignocellulose (NDF > 65 % DM) and lignin (ADL = 15 % DM) contents. DK are characterised by high levels of lignocellulose (NDF = 67.7 % DM) and a low level of lignin (ADL = 4.2 % DM) contents. WD are characterised by high levels of nitrogen-free extract (72.9 % DM) and total sugars (49.0 % DM) and a low level of lignocellulose contents (NDF = 13.3 % DM). Date palm by-products contained a low protein concentration ranging from 2.5 % to 5.8 % DM (Table 1).

**Table 1:** Dry matter (%) and chemical composition (% DM) of date palm by-products.

	Floral stems	Palm fronds	Date kernels	Wasted dates
Dry matter${}^{*}$	44.7	54.4	89.4	86.0
Organic matter	92.3	91.3	98.7	97.7
Crude protein	2.5	4.2	5.8	3.4
Ether extracts	1.3	2.2	7.3	1.8
Crude fibre	43.0	40.0	30.0	6.0
Neutral detergent fibre	71.7	65.8	67.7	13.3
Acid detergent fibre	43.5	40.3	31.9	7.7
Acid detergent lignin	15.0	15.1	4.2	2.2
Hemicellulose	28.2	25.5	35.8	6.6
Cellulose	28.5	25.2	27.7	5.2
Ash	7.7	8.7	1.3	2.3
Phosphorus	0.03	0.04	0.10	0.04
Calcium	0.07	0.73	0.04	0.28
Condensed tannin	2.4	2.2	1.84	1.65
Nitrogen-free extract	16.8	19.1	17.9	79.2
Total sugars	–	–	–	49.0

${}^{*}$ % fresh mass.

### 3.2 Enzymatic activity

This exogenous fibrolytic enzyme contains 1180 units of xylanase g${}^{-1}$, 750 units of endoglucanase g${}^{-1}$, and 740 units of exoglucanase g${}^{-1}$.

### 3.3 Pre-incubation effects

Adding EFE to FS, PF, and DK did not affect the OM solubility, but it tended to increase the soluble organic matter of WD. With the highest dose, the OM solubility increased by 6.6 % (Table 2).

**Table 2:** Effect of the dose of EFE (mg (g DM)${}^{-1})$ on the soluble organic matter after 12 h of pre-incubation of date palm by-products (%).

D	FS	PF	DK	WD
0	16.1	16.6	10.4	48.3
0.5	16.1	17.6	10.2	48.3
1	16.9	16.8	9.5	49.9
2	15.6	16.4	9.5	51.5
Linear	0.82	0.27	0.29	0.07
Quadratic	0.47	0.15	0.60	0.86
SEM	1.4			
P values				
S	< 0.001			
D	0.3			
S × D	0.46			

DK is the date kernels; WD is the wasted dates; FS is the floral stems; PF is the palm fronds; S is the substrate; SEM is standard error of the mean; D is dose; S × D is the interaction between dose and substrate; L is the linear effect; Q is the quadratic effect.

### 3.4 The in vitro gas production

The cumulative gas production of WD was significantly greater than that of PF, DK, and FS. As for the gas production parameters, WD had the highest potential gas production, the fastest gas production rate, and the shortest time which the gas production was half the potential gas production. PF and FS showed the lowest potential gas production, but DK had the lowest gas production rate (Table 3). The addition of EFE to DK quadratically affected the gas production from 48 to 96 h and the potential gas production, especially with the lowest enzyme dose. In fact, the potential gas production increased by 14.8 %, but the gas production rate and the time which the gas production was half the potential gas production were unaffected. For WD, adding EFE tended to linearly increase the gas production rate and gas production during the first 4 h of incubation. But it tended to decrease the time which the gas production was half the potential gas production without affecting the potential gas production. For FS and PF, the addition of EEF did not affect cumulative gas production and gas production parameters.

**Table 3:** Effect of increasing the dose of EFE (mg (g DM)${}^{-1}$) on gas production parameters and cumulative gas production of date palm by-products.

S	D	Gas production parameters			Cumulative gas production (mL (g DM)${}^{-1}$) at								
		B	C	$T_{1/2}$	2 h	4 h	6 h	8 h	12 h	24 h	48 h	72 h	96 h
FS	0	112.4	0.092	7.53	29.1	45.6	55.0	61.0	68.3	82.0	101.5	119.1	126.5
	0.5	106.9	0.092	7.69	29.4	43.7	52.1	56.7	63.8	78.0	98.4	112.4	119.8
	1	109.0	0.082	8.61	28.0	41.0	49.5	54.9	62.5	78.0	96.7	113.7	120.4
	2	108.8	0.078	8.99	28.3	41.2	49.2	54.0	61.3	76.1	96.2	114.0	120.8
	L	0.24	0.10	0.16	0.41	0.05	0.05	0.05	0.06	0.12	0.13	0.19	0.12
	Q	0.15	0.69	0.88	0.78	0.46	0.24	0.27	0.31	0.47	0.49	0.1	0.06
PF	0	125.6	0.105	6.84	30.4	52.4	65.6	70.8	80.6	102.8	117.2	129.0	139.4
	0.5	118.8	0.090	7.82	32.4	46.9	56.0	60.6	71.8	92.6	108.3	121.6	132.9
	1	127.4	0.087	7.99	35.4	50.5	61.0	65.1	71.0	95.2	114.2	130.9	144.8
	2	126.3	0.083	8.34	33.1	48.4	59.2	63.6	69.6	93.5	111.4	130.1	143.5
	L	0.32	0.170	0.30	0.62	0.69	0.56	0.45	0.32	0.34	0.65	0.54	0.29
	Q	0.84	0.59	0.67	0.50	0.75	0.48	0.47	0.31	0.40	0.64	0.74	0.96
DK	0	234.1${}^{b}$	0.033	21.83	21.8	34.3	41.8	48.9	65.1	123.9	196.2	211.2${}^{b}$	216.1${}^{b}$
	0.5	268.7${}^{a}$	0.030	24.29	21.5	32.7	39.7	45.8	65.3	141.2	213.7	239.2${}^{a}$	237.7${}^{a}$
	1	241.8${}^{b}$	0.031	23.22	23.5	33.6	40.3	47.0	62.5	131.2	202.2	220.7${}^{b}$	225.6${}^{b}$
	2	242.5${}^{b}$	0.031	22.43	21.3	32.4	39.7	46.9	62.7	130.6	200.1	218.7${}^{b}$	223.6${}^{b}$
	L	0.66	0.51	0.86	0.92	0.56	0.59	0.79	0.71	0.74	0.82	0.84	0.83
	Q	0.01	0.150	0.09	0.39	0.91	0.73	0.64	0.91	0.09	0.07	0.01	0.03
WD	0	283.2	0.159	4.37	102.1	151.0	180.1	200.0	223.9	255.7	287.0	300.8	306.8
	0.5	287.5	0.162	4.28	97.3	154.0	181.9	201.1	224.1	255.5	287.8	300.1	306.2
	1	292.3	0.167	4.16	101.8	158.8	189.4	208.5	230.6	262.6	293.9	305.4	312.5
	2	284.9	0.169	4.10	113.1	171.5	191.3	209.1	230.7	257.9	288.8	300.9	306.6
	L	0.57	0.08	0.08	0.07	0.08	0.42	0.53	0.65	0.85	0.88	0.96	0.95
	Q	0.43	0.32	0.28	0.18	0.79	0.84	0.85	0.89	0.77	0.75	0.83	0.78
SEM		19.2	0.01	2.76	4.9	6.3	8.3	13.1	14.9	13.6	13.55	14.6	14.8
P values													
S		< 0.001	< 0.001	< 0.001	< 0.001	< 0.001	< 0.001	< 0.001	< 0.001	< 0.001	< 0.001	< 0.001	< 0.001
D		0.88	0.14	0.69	0.64	0.57	0.43	0.14	0.41	0.96	0.87	0.90	0.88
S × D		0.76	0.59	0.96	0.88	0.58	0.54	0.27	0.72	0.99	0.65	0.61	0.39

${}^{a,b}$ Means of doses for each variable that do not have a common superscript differ at P <0.05. B is the potential gas production (mL (g DM)${}^{-1})$. C is the gas production rate (mL (h)${}^{-1})$. $T_{1/2}$ is a time which the gas production is half the potential gas production (h). DK is the date kernels. WD is the wasted dates; FS is the floral stems. PF is the palm fronds. S is substrate. D is the dose. S × D is the interaction between dose and substrate. L is the linear effect. Q is the quadratic effect.

### 3.5 The in vitro rumen fermentation profile

WD has the highest OMD, EM, and VFA. Enzymatic supplementation tended to increase the OMD, ME, and VFA only for DK with the lowest enzyme dose (7.8 %, 8.4 % and 13.9 % respectively (Table 4)).

**Table 4:** Effect of increasing the dose of EFE (mg (g DM)${}^{-1})$ on in vitro rumen fermentation profiles of date palm by-products.

S	D	OMD	ME	VFA
FS	0	31.1	4.6	0.360
	0.5	30.4	4.5	0.342
	1	30.3	4.5	0.341
	2	30.0	4.4	0.334
	L	0.12	0.12	0.12
	Q	0.47	0.47	0.47
PF	0	35.6	5.2	0.452
	0.5	33.8	5.0	0.406
	1	34.3	5.0	0.418
	2	34	5.0	0.411
	L	0.34	0.34	0.34
	Q	0.40	0.40	0.40
DK	0	39.6	5.9	0.546
	0.5	42.7	6.4	0.622
	1	40.9	6.1	0.578
	2	40.8	6.1	0.576
	L	0.74	0.74	0.74
	Q	0.09	0.09	0.09
WD	0	60.6	9.4	1.131
	0.5	59.2	9.4	1.141
	1	61.9	9.5	1.162
	2	60.6	9.3	1.130
	L	0.85	0.85	0.85
	Q	0.77	0.77	0.77
SEM		3.94	0.06	0.09
P values				
S		<0.001	<0.001	<0.001
D		0.96	0.96	0.96
S × D		0.99	0.99	0.99

ME is metabolisable energy, OMD is organic-matter degradability ( %), ME is metabolisable energy (MJ (kg DM)${}^{-1}$), VFA is volatile fatty acids (mmol (200 mg DM)${}^{-1}$), DK is the date kernels, WD is the wasted dates, FS is the floral stems, PF is the palm fronds, S is the substrate, D is dose, S × D is the interaction between dose and substrate, L is the linear effect, and Q is the quadratic effect.

## 4 Discussion

The chemical compositions of date palm by-products in this study are similar to the results found by Genin et al. (2004), Kholif et al. (2015), and Boufennara et al. (2016). The CP content of date palm by-products was generally low (CP < 6 % DM) especially for floral stems (CP = 2.5 % DM). This CP content was under the level required (7 % DM–8 % DM) for optimum rumen function (Van Soest, 1994). The WD has the highest gas volume, gas production rate, OMD, ME, and VFA. This could be due to their high level of total sugars, while the FS and the PF have the lowest cumulative gas production and fermentation profiles. This could be due to their high level of fibre, especially the lignin fraction, which decreases the attachment of ruminal microbes to feeds (Paya et al., 2007), and to their high condensed tannins which partially inhibit the activity of rumen flora (Williams and Coleman, 1991).

The responses to EFE addition are influenced by the by-products structures. These results are similar to Elghandour et al. (2013), who showed that the fibrolytic enzymes are most effective for *Sorghum vulgare* (straw), followed by *Andropogon gayanus* (leaves) and *Pennisetum purpureum* (leaves), and lastly *Saccharum officinarum* (leaves). For DK, EFE increased the potential gas production and tended to increase the OMD, ME, and VFA with the lowest dose. Similarly, Kholif et al. (2015) found that the treatment of DK with commercial cellulolytic enzymes (Veta-Zyme Plus^®^ produced by Vetagri^®^ Consulting Inc, Canada; contains 400 units of cellulose g${}^{-1}$, 550 units of amylase g${}^{-1}$, 2000 units of protease g${}^{-1}$) increased DM and OM digestibility in vitro. In addition, they showed that the lowest enzyme dose was more effective. This feed additive increased the volume of gas at the end of incubation. This indicates that the exogenous fibrolytic enzymes increased the fermentable material. Increasing the gas production without improving the soluble organic matter in the pre-incubation period is consistent with the result found by Colombatto et al. (2003) when they treated alfalfa stems with a commercial fibrolytic enzyme (Liquicell 2500; Specialty Enzymes and Biochemicals Co. CA, USA). Those results indicated that the exogenous fibrolytic enzymes act synergistically with ruminal microbial enzymes (Morgavi et al., 2000) and the enzymes may also change the fibrolytic structure of the DK during the period of substrate–enzyme interaction, making them more amenable to rumen microorganism attachment (Nsereko et al., 2000). For WD, the addition of EFE tended to increase the gas production rate of fermentation and gas production at the beginning of incubation, at the highest dose. Otherwise, it tended to decrease the time which the gas production was half the potential gas production, with the same dose. But it did not affect the potential gas production and the gas produced at the end of incubation. Similarly, Giraldo et al. (2008) and Ranilla et al. (2008) reported that the effects of enzyme became less marked as incubation time progresses. This enhancement of gas production at the beginning of incubation might be due to the increased soluble organic matter in the interaction period (Table 2) and to the release of soluble carbohydrates (Nsereko et al., 2000). This change provides additional energy for the development of bacteria and shortens the time of colonisation to feed (Yang et al., 1999; Wang et al., 2001). The increase in fermentation, measured at an early stage of in vitro incubation, would be beneficial in vivo because it is an indicator of the efficiency of ingestion, at which feedstuffs are used in vivo (Wang et al., 2013). For FS and PF, the addition of EFE did not affect the soluble organic matter in the interaction period, the cumulative gas production at any incubation time, gas production parameters, and the rumen fermentation profile. The EFE effect could be imperfect due to the lack of enzymes, which solubilise the lignin–cellulose structure, which prevents access of fibrolytic enzymes to cellulose and hemicelluloses (Hatfield et al., 1999). Also, it is imperfect due to the lack of an enzyme that hydrolyses tannin substrates which partially inhibit the activity of digestive enzymes (Molina-Alcaide et al., 2008). Abd El Tawab et al. (2016) found that adding cellulase and tannase enzymes to feed diets containing palm fronds plays a major role in increasing the digestibility. This commercial exogenous fibrolytic enzyme can exhibit inhibitory effects at higher doses. The non-linear effects of enzyme doses have been also proved in vivo (Lewis et al., 1999; Kung et al., 2000) and in vitro (Diaz et al., 2013). Several hypotheses explained these results such as Nsereko et al. (2000), who hypothesised that the free hydrolysed sugar, which remains bound to the fibre, could block the sites of action of the enzyme (like glucan hydrolases). Morgavi et al. (2004) hypothesised that excessive doses of enzyme block the adhesion sites for the bacteria, which decreases the microbial adhesion to the substrates. Treacher and Hunt (1996) also proved that the high dose of exogenous fibrolytic enzymes can release anti-nutritional factors, such as phenolic compounds.

## 5 Conclusion

It can be concluded that the effectiveness of the exogenous fibrolytic enzyme was influenced by the chemical composition of the date palm by-products and the dose of the enzymes. This feed additive especially increased ruminal fermentation of date kernels at the lowest dose.

## Data Availability

No data sets were used in this article.
